# The Rapid Inactivation of Porcine Skin by Applying High Hydrostatic Pressure without Damaging the Extracellular Matrix

**DOI:** 10.1155/2015/587247

**Published:** 2015-03-24

**Authors:** Naoki Morimoto, Atsushi Mahara, Kouji Shima, Mami Ogawa, Chizuru Jinno, Natsuko Kakudo, Kenji Kusumoto, Toshia Fujisato, Shigehiko Suzuki, Tetsuji Yamaoka

**Affiliations:** ^1^Department of Plastic and Reconstructive Surgery, Kansai Medical University, 2-5-1 Shinmachi, Hirakata, Osaka 573-1010, Japan; ^2^Department of Biomedical Engineering, National Cerebral and Cardiovascular Center Research Institute, Fujishirodai, Suita, Osaka 565-8565, Japan; ^3^Department of Biomedical Engineering, Osaka Institute of Technology, 5-16-1 Omiya, Asahi-ku, Osaka 535-8585, Japan; ^4^Department of Plastic and Reconstructive Surgery, Graduate School of Medicine, Kyoto University, Kyoto 606-8507, Japan

## Abstract

We previously reported that high hydrostatic pressure (HHP) of 200 MPa for 10 minutes could induce cell killing. In this study, we explored whether HHP at 200 MPa or HHP at lower pressure, in combination with hyposmotic distilled water (DW), could inactivate the skin, as well as cultured cells. We investigated the inactivation of porcine skin samples 4 mm in diameter. They were immersed in either a normal saline solution (NSS) or DW, and then were pressurized at 100 and 200 MPa for 5, 10, 30, or 60 min. Next, we explored the inactivation of specimens punched out from the pressurized skin 10 × 2 cm in size. The viability was evaluated using a WST-8 assay and an outgrowth culture. The histology of specimens was analyzed histologically. The mitochondrial activity was inactivated after the pressurization at 200 MPa in both experiments, and no outgrowth was observed after the pressurization at 200 MPa. The arrangement and proportion of the dermal collagen fibers or the elastin fibers were not adversely affected after the pressurization at 200 MPa for up to 60 minutes. This study showed that a HHP at 200 MPa for 10 min could inactivate the skin without damaging the dermal matrix.

## 1. Introduction

Various kinds of skin substitutes, such as biosynthetic skin substitutes, allografts, xenografts, and bioengineered skin substitutes, which include human cells, have been developed and used for the treatment of burns, chronic ulcers, and other wounds [1–4].

Recently, decellularized matrices have been used and reported to be useful in tissue regeneration and also in skin regeneration [5–8]. These acellular dermal matrices are distinctly different from the simple collagen scaffolds and can provide temporary mechanical strength to the repair site [8, 9]. The decellularization process usually consists of two steps. The first step is a process of tissue inactivation and the second is the subsequent washing process to the remove cellular debris [8, 9]. The second step is fairly uniform, but a variety of techniques that use chemical agents such as acid, sodium dodecyl sulfate (SDS), or Triton X; biological agents such as trypsin, collagenase, nucleases, or dispase; and physical methods such as freeze-thaw, hydrostatic pressure, or electroporation have been reported for use in the first step [6, 9, 10]. The first process is selected depending on the cellularity, density, and thickness of the target tissue in order to avoid damaging the extracellular matrix and its native structure. This process usually takes several days, which can restrict the potential for clinical application.

We previously reported that high hydrostatic pressure (HHP) of more than 600 MPa for 10 minutes could destroy cell membranes uniformly and in a short treatment time (within one hour, including the increasing and decreasing process of pressure), regardless of the thickness or hardness of the tissue [11–13]. Tissues were packed in a plastic bag that was filled with a normal saline solution (NSS) during the pressurization process in our procedure. Next, we investigated the minimum pressure for cell killing using mammalian cultured cells and we reported that hydrostatic pressure of 200 MPa for only 10 minutes could induce cell killing through the inactivation of mitochondrial activity [14]. In this study, we explored whether these pressure conditions of 200 MPa for 10 min could inactivate porcine skin, which has a similar structure to human skin, as well as cultured cells. We also evaluated the possibility of inactivating cells with the lower pressure than 200 MPa by using distilled water that was hyposmotic and could inactivate cells easily compared to the NSS or using a longer pressurization time with a maximum of 60 min. In addition, we also evaluated the damage of the extra cellular matrix of the skin according to the maximum pressurization time of 60 minutes. Then, we discussed the possibility of clinical applications by using the inactivated skin produced by the HHP for skin regeneration.

## 2. Materials and Methods

### 2.1. Ethics Statement

All animal experiments were conducted in accordance with the Guidelines for Animal Experiments established by the Ministry of Health, Labour, and Welfare of Japan and by the National Cerebral and Cardiovascular Center Research Institute, Japan. Our protocol was approved by the Committee on the Ethics of Animal Experiments of the National Cerebral and Cardiovascular Center Research (Permit Number: 009017).

### 2.2. Preparation of Porcine Skin

One-year-old male Göttingen Minipigs (Ellegaard, Dalmose, Denmark) (*n* = 3) were obtained and maintained on a standard diet of commercially available pellets that did not contain any medication and water was given ad libitum. The skin was resected from the abdominal region under general anesthesia using 1% propofol (Diprivan: AstraZeneca K.K., Osaka, Japan) after intubation and the skin defect was sutured using 3-0 nylon (Ethicon, Somerville, NJ). Subcutaneous tissues were removed from the skin using scissors and the full-thickness of the skin was used in the experiments. Skin specimens that measured 4 mm in diameter and used in the first experiment were taken from the first minipig and skin specimens that measured 10 × 2 cm in size and used in the second experiment were taken from the second minipig. Gross photos of the skin specimens and the HE, Azan, and EVG (Elastica van Gieson) staining of the specimens were performed using the skin taken from the third minipig.

### 2.3. Pressurization of the Skin

In the first experiment, skin specimens (*n* = 68) that measured 4 mm in diameter were punched out using biopsy punches (Kai Industries Co., Ltd., Tokyo, Japan) and were pressurized using a cold isostatic pressurization machine (Dr. Chef; Kobelco, Japan). After packing the specimens in plastic bags filled with a normal saline solution (NSS; Otsuka Pharmaceutical Ltd., Tokyo, Japan) or distilled water (DW; Otsuka Pharmaceutical Ltd., Tokyo, Japan), the bags were immersed in the transmission fluid in the sample chamber of the machine. The atmosphere inside of the sample chamber was pressurized at a rate of 65.3 MPa/min until the target pressure of 100 or 200 MPa was reached [11, 14]. The target pressure was maintained for 5, 10, 30, or 60 minutes and then decreased to atmospheric pressure at the same rate. Skin specimens that were preserved in the NSS without the pressurization were used as a control.

In the next step, we first pressurized large skin samples and evaluated the viability of the punched-out skin samples after pressurization. Skin specimens that measured 10 × 2 cm in size and immersed in the NSS or DW were preserved without pressurization or pressurized at 100 and 200 MPa for 10 minutes in the same manner. After pressurization, the skin specimens that measured 8 mm in diameter or 4 mm in diameter (*n* = 4 in each group) were punched out using biopsy punches and used in the following evaluation.

### 2.4. Evaluation of the Viability of the Pressurized Skin

The viability of the pressurized skin was evaluated using the WST-8 (4-[3-(2-methoxy-4-nitrophenyl)-2-[4-nitrophenyl]-2H-5-tetrazolio]-1,3-benzene disulfonate sodium salt) assay and an explant outgrowth culture.

#### 2.4.1. WST-8 Assay

The WST-8 assay evaluates the mitochondrial enzyme activity in cells. In the first experiment, samples of the unpressurized skin (*n* = 4) and the pressurized skin (*n* = 4 in each group) that measured 4 mm in diameter were pressurized for 5, 10, 30, or 60 minutes in the NSS or DW and placed into wells of a 96-well plate (AGC TECHNO GLASS CO. Ltd., Tokyo, Japan), and 100 *μ*L of the DMEM (Dulbecco's Modified Eagle Medium; Life Technologies Japan Ltd., Tokyo, Japan) was added to each well, and these samples were incubated at 37°C for 15 minutes. Then, 10 *μ*L of the WST-8 assay reagent (Dojindo Laboratories, Kumamoto, Japan) was added to each well and incubated at 37°C for 1 hour. Subsequently, the plate was gently shaken and the absorbance at 450 nm was measured using a multiplate reader (Thermo Varioskan Flash; Thermo Fisher Scientific Inc., MA, USA). The absorbance of the DMEM in the vacant wells (*n* = 43) was also measured and this absorbance was used as an arbitrary zero point.

In the second experiment used skin specimens that measured 10 × 2 cm in size and skin specimens that measured 8 mm in diameter (*n* = 4 in each group) were punched out of the skin that was preserved in either the NSS or DW without pressurization or the pressurized skin that was in NSS or DW and pressurized at 100 and 200 MPa. Each specimen was put in a 2.0 mL microtube (Watson Co., Ltd., Tokyo, Japan) and 200 *μ*L of the DMEM was added to each tube and incubated at 37°C for 15 minutes. Then, 20 *μ*L of the WST-8 assay reagent was added to each well and incubated at 37°C for 1 hour. Subsequently, 50 *μ*L of the solution was transferred to each well of a 96-well plate and the absorbance at 450 nm was measured. The absorbance of the DMEM (*n* = 4) was used as an arbitrary zero point.

#### 2.4.2. Outgrowth Culture of Pressurized Skin

In the second experiment, skin specimens that measured 4 mm in diameter (*n* = 4 in each group) were punched out from the pressurized skin in the NSS or DW and pressurized at 100 and 200 MPa. Each specimen was placed in a well in a 24-well plate (AGC TECHNO GLASS CO. Ltd., Tokyo, Japan). A small piece of slide glass was placed on each specimen to prevent it from floating. One milliliter of the DMEM with 10% FBS was added and cultured in humidified 95% air and 5% CO_2_ at 37°C. The culture medium was changed every three days. The pressurized skin and slide glasses were removed on Day 14. Micrographs of the cells were taken and the numbers of outgrowth cells were compared using the WST-8 assay on Day 21. A total of 100 *μ*L of the DMEM was added to each well and incubated for 15 minutes, after which 10 *μ*L of the WST-8 assay reagent was added to each well and incubated at 37°C for 1 hour. Then, 50 *μ*L of the solution was transferred to each well of a 96-well plate and the absorbance at 450 nm was measured. The absorbance of the DMEM (*n* = 4) was used as an arbitrary zero point.

### 2.5. Evaluation of the Histology of the Pressurized Skin

Gross photographs were taken of the pressurized skin samples or the samples of a square that measured 15 mm in size from each side of the third minipig and pressurized at 100 and 200 MPa for 10 minutes in the NSS or DW. Next, the skin samples were fixed with a 10% neutral-buffered formalin solution and embedded in paraffin blocks. The central area of each sample was sectioned at a 5 *μ*m thickness and subjected to hematoxylin and eosin (HE) staining, Azan staining, and EVG (Elastica von Gieson) staining.

Dermal collagen fibers of pressurized skin were also examined by scanning electron microscopy (SEM). Specimens from the first minipig were fixed in 2% glutaraldehyde and dehydrated in a graded series of ethanol from 50% to 100%. They were dried in a* t*-butanol dryer, coated with platinum, and observed by SEM (JSM-6390LV, JEOL Ltd., Tokyo, Japan).

### 2.6. Evaluation of the Elasticity of the Pressurized Skin

The young modulus of pressurized skin was calculated from compression stress-strain measurements. Another skin specimen (*n* = 1) from the third minipig of a square that was 15 mm in size for each side was put on the stage of a rheometer (MCR 301, Anton Par, Austria). Then, each specimen was compressed with the speed of 10 *μ*m/sec three times for each specimen and the initial compressive curves were analyzed.

### 2.7. Statistical Analysis

All data are expressed as the means ± standard error (SE). The Kruskal–Wallis test, followed by the Steel–Dwass test, was used for statistical analysis. The Microsoft Excel software program and the Statcel software add-on (OMS Publishing, Inc., Tokyo, Japan) was used for all statistical analyses and *P* < 0.05 was accepted as significant.

## 3. Results

### 3.1. The Mitochondrial Enzyme Activity of the Pressurized Skin (WST-8 Assay)

**Figure 1:**
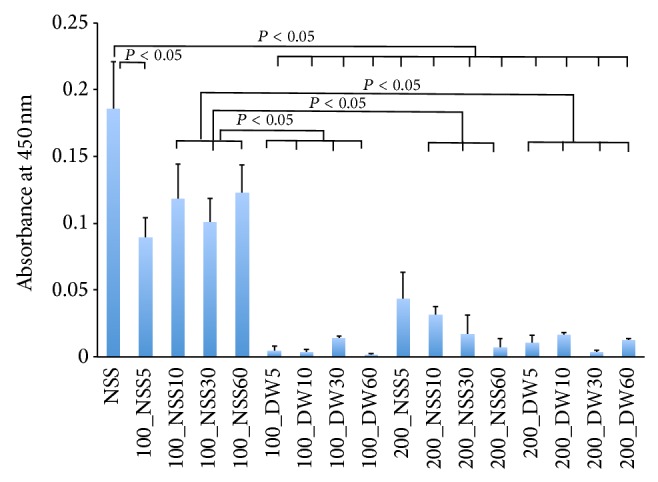
Quantification of the mitochondrial activity of the pressurized skin measured by the WST-8 assay. The horizontal axis shows the pressure, solution, and pressurization time in order. NSS shows the absorbance of the skin that was preserved in the NSS without pressurization. 100_NSS5 means the skin immersed in NSS pressurized at 100 MPa for 5 minutes and 100_DW5 means the skin immersed in DW pressurized at 100 MPa for 5 minutes. A significant difference is shown in the figure. The absorbance was significantly decreased in the skin immersed in the DW at the pressure of 100 MPa, and in the NSS and the DW at the pressure of 200 MPa, compared with that in NSS without pressurization (*P* < 0.05). The absorbance for the skin in the NSS at the pressure of 200 MPa for 10, 30, and 60 min and in the DW at the pressure of 200 MPa was significantly decreased compared with that in NSS at 100 MPa for 10, 30, and 60 min (*P* < 0.05). The activity of the skin immersed in NSS at the pressure of 200 MPa and in DW at the pressure of 100 and 200 MPa showed no activity level.

The mitochondrial enzyme activity of the pressurized skin that measured 4 mm in diameter in the first experiment is shown in Figure 1. As for the pressurization time, there was no significant difference in the absorbance level among the pressurization times of 5, 10, 30, and 60 min for the same pressurization group at both 100 and 200 MPa. The absorbance levels for the skin that was in NSS and pressurized at 200 MPa for 10, 30, and 60 min and in DW and pressurized at 100 MPa and 200 MPa were significantly decreased compared to those in the NSS and pressurized at 100 MPa for 10, 30, and 60 min (*P* < 0.05). The absorbance of the skin in the NSS and pressurized at 200 MPa and in DW and pressurized at 100 and 200 MPa showed no mitochondrial enzyme activity. These findings suggest that the mitochondrial enzyme activities of the pressurized skin that was punched out before the pressurization were inactivated in the NSS at the 200 MPa pressure for 10, 30, and 60 min and in the DW at the pressure of 100 MPa and 200 MPa.

**Figure 2:**
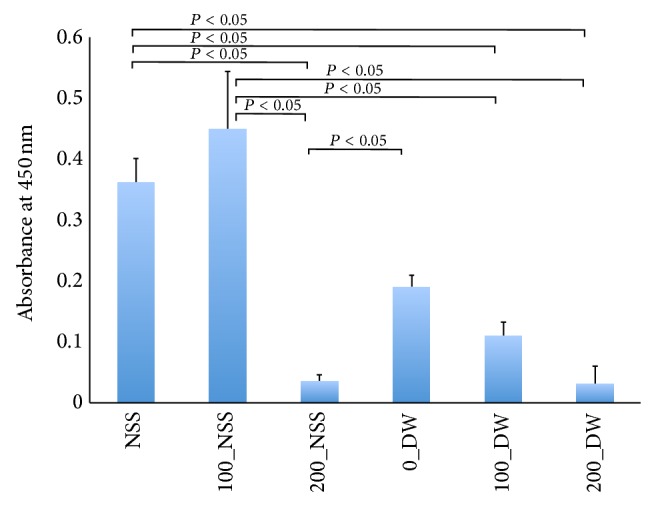
Quantification of mitochondrial activity of punched-out skin from the pressurized skin measured by a WST-8 assay. Horizontal axis shows the pressure and solution in order. The NSS shows the absorbance of the skin preserved in the NSS without pressurization. 100_NSS means the skin sample was punched out from the skin and immersed in NSS and pressurized at 100 MPa and 0_DW means the skin was punched out from the skin and immersed in DW without pressurization. A significant difference is shown in the figure. The absorbance of 200_NSS was decreased compared with those of NSS, 100_NSS, and 0_DW (*P* < 0.05). The absorbance levels of 100_DW and 200_DW were significantly lower than those of NSS and 100_NSS (*P* < 0.05). The absorbance levels of 200_NSS and 200_DW showed no activity level.

The mitochondrial enzyme activities of the skin samples that measured 8 mm in diameter and were punched out from pressurized skin for 10 min in the second experiment are shown in Figure 2. The absorbance levels of the 200_NSS and 200_DW decreased and showed no activity level. The absorbance of the 100_DW was significantly lower than that of the NSS and 100_NSS (*P* < 0.05), but it showed some activity. These findings suggest that mitochondrial enzymes were inactivated in the NSS and DW at the pressure of 200 MPa.

### 3.2. Outgrowth Culture from the Pressurized Skin

**Figure 3:**
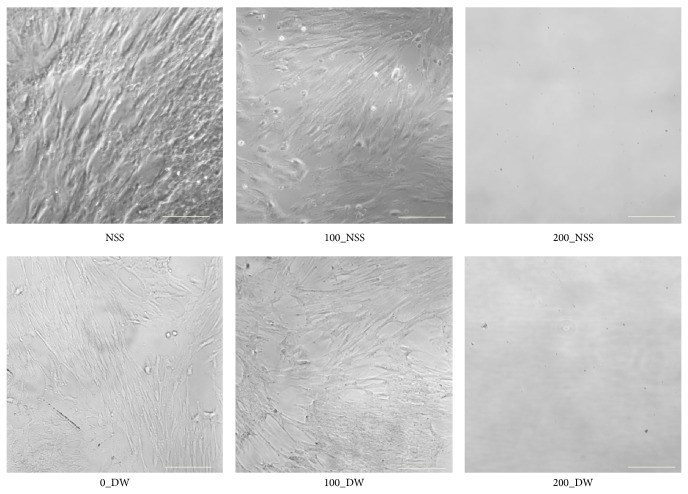
Micrographs of the outgrowth fibroblasts on Day 21. Fibroblasts were observed in NSS, 100_NSS, 0_DW, and 100_DW. No outgrowth cells were confirmed in 200_NSS and DW-200. Scale bar: 100 *μ*m.

**Figure 4:**
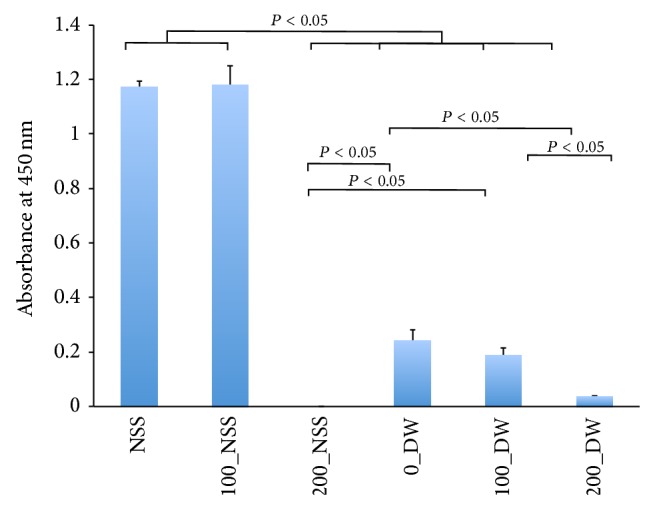
Quantification of the mitochondrial activity from the outgrowth fibroblasts from punched-out pressurized skin. The horizontal axis shows the pressure and solution in order, as shown in Figure 2. The absorbance levels of 200_NSS, 0_DW, 100_DW, and 200_DW were significantly lower than those of NSS and 100_NSS (*P* < 0.05). The absorbance of 200_NSS was significantly lower than that of 0_DW and 100_DW (*P* < 0.05), and that of 200_DW was significantly lower than that of 0_DW (*P* < 0.05) and 100_DW (*P* < 0.05).

In the second experiment, the outgrowth of fibroblasts was confirmed in the NSS, 100_NSS, 0_DW, and 100_DW; however, no outgrowth of fibroblasts was observed in the 200_NSS and 200_DW (Figure 3). The mitochondrial enzyme activities of outgrowth fibroblasts from the punched-out pressurized skin are shown in Figure 4. The absorbance levels of the 200_NSS and the 200_DW were significantly lower than the others and showed no activity level. These findings suggest that the skin was inactivated after pressurization at 200 MPa, regardless of the NSS or DW, and the fibroblasts were not viable and could not proliferate in culture medium. Fibroblasts that grew from the skin in the DW at the pressure of 100 MPa for 10 min showed lower WST-8 activity in the second experiment. These findings suggest that the cells on the superficial part of the skin that were immersed in the DW were inactivated according to the low osmotic pressure of DW as shown in the WST-8 assay in the first experiment. However, the cells in the deeper part were not inactivated by DW or pressurized at 100 MPa and proliferated; therefore, pressurization at 200 MPa was needed to achieve the complete inactivation of the cells in skin specimens.

### 3.3. Histological Evaluation of Pressurized Skin

**Figure 5:**
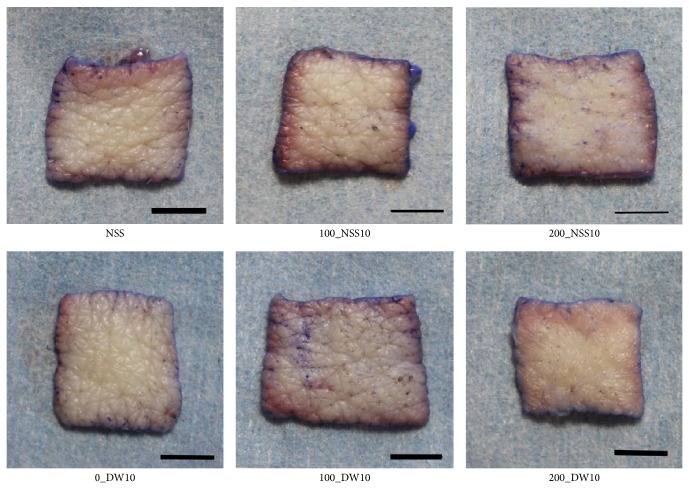
Gross appearance of the pressurized skin. The gross appearance of the skin specimens in NSS, 100_NSS, 200_NSS, 0_DW, 100_DW, and 200_DW was shown. The dermatoglyphics in 200_NSS and 200_DW were not clear. Scale bar: 5 mm.

**Figure 6:**
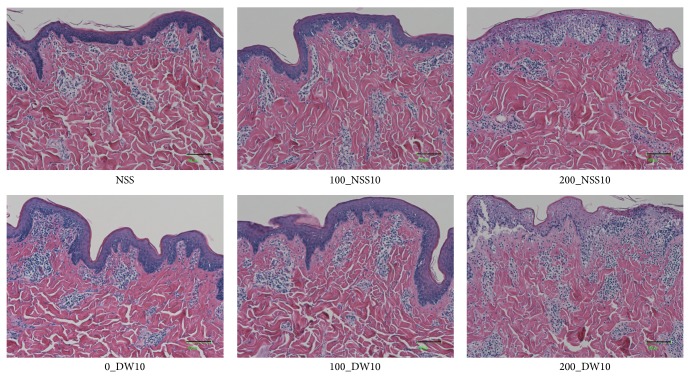
The micrographs of the HE stained pressurized skin. The micrographs of the HE stained pressurized skin in NSS, 100_NSS10, 200_NSS10, 100_DW10, and 200_DW10 are shown. The epidermis was intact after pressurization at 100 MPa. On the other hand, the epidermis was vacuolated and to be detached in 200_NSS10 and 200_DW10. Scale bar: 100 *μ*m.

**Figure 7:**
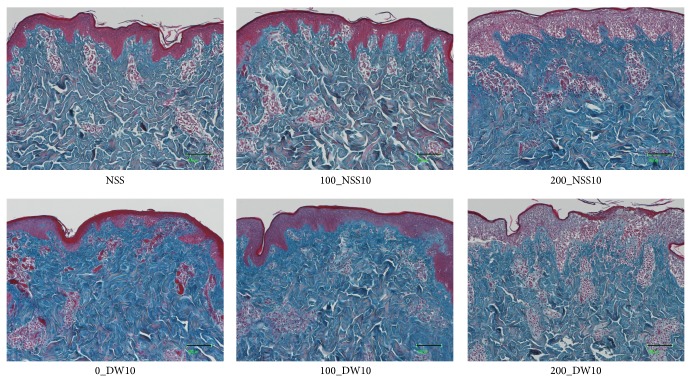
Micrographs of the Azan stained pressurized skin. The micrographs of the Azan stained pressurized skin in NSS, 100_NSS10, 200_NSS10, 100_DW10, and 200_DW10 are shown. The degeneration of dermal collagen fiber was not observed in all specimens. Scale bar: 100 *μ*m.

**Figure 8:**
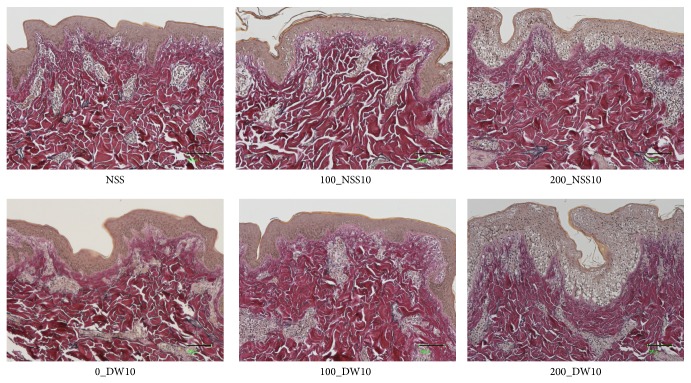
Micrographs of the EVG (Elastica van Gieson stain) pressurized skin. The micrographs of the EVG stained pressurized skin in NSS, 100_NSS10, 200_NSS10, 100_DW10, and 200_DW10 are shown. Degeneration of dermal collagen fiber and elastin fibers was not observed in all specimens. Scale bar: 100 *μ*m.

Gross photos of each specimen were shown and no obvious changes were observed in 0_DW, 100_NSS, and 100_DW (Figure 5). Dermatoglyphics in the 200_NSS and 200_DW samples were not clear; however, this is because of the detachment of the epidermis as shown in the HE sections in Figure 6. Skin specimens without pressurization and those pressurized at 100 MPa had an intact epidermis, regardless of the use of NSS or DW in HE sections (Figure 6). However, the epidermal cells of the skin specimens that were pressurized at 200 MPa in the NSS and DW were vacuolated to be removed from the dermis. Degeneration of the dermal collagen fiber was not observed in all of the specimens in the Azan stained (Figure 7) and EVG stained sections (Figure 8). Elastic fibers were also not degenerated in all of the specimens in the EVG stained sections (Figure 8).

**Figure 9:**
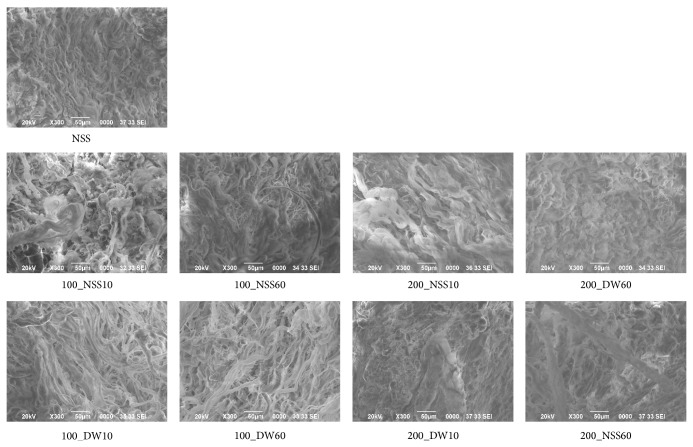
The SEM micrographs of pressurized skin. The SEM micrographs of the dermis in NSS, 100_NSS10, 100_NSS60, 200_NSS10, 200_NSS60, 100_DW10, 100_DW60, 200_DW10, and 200_DW60 are shown. The arrangement and proportion of collagen fibers were close enough and not adversely affected after pressurization, regardless of the processing time. Scale bar: 50 *μ*m.

The SEM micrographs of the dermis of each specimen are shown in Figure 9. The arrangement and proportion of the dermal collagen fibers were not adversely affected after pressurization at 100 and 200 MPa for up to 60 min.

### 3.4. Elasticity of the Pressurized Skin

**Figure 10:**
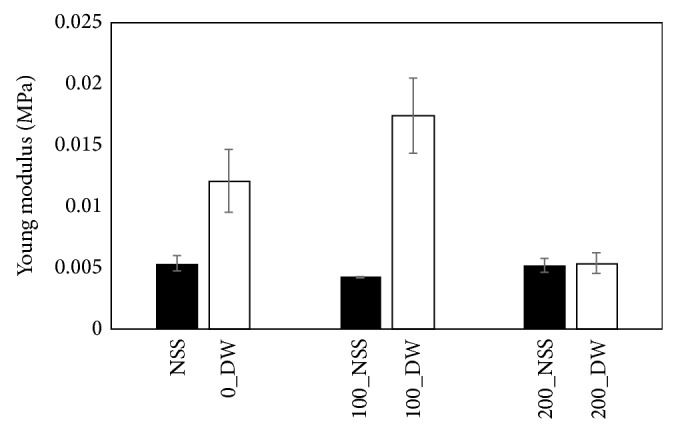
Comparison of the elasticity of the pressurized skin. The young modulus of the pressurized skin in DW tended to be high especially at 100 MPa. However, the young modulus in NSS groups was not changed after pressurization at 100 and 200 MPa.

The young modulus of the pressurized skin in the DW tended to be high especially at the pressure of 100 MPa (Figure 10). However, the young modulus in the NSS groups was not changed after pressurization at 100 and 200 MPa.

## 4. Discussion

In this study, we explored the possibility of applying a HHP for the inactivation of skin. Although various methods have been reported to inactivate soft and hard tissue, it is still difficult to inactivate tissue rapidly without damaging the extracellular matrix (ECM) [6]. In terms of damage to the ECM, chemical agents or biological agents have the possibility of damaging it or causing residual toxicity to the target tissue. We reported that SDS had a superior decellularization ability compared to chemical or biological agents, but the inactivation of the dermis by SDS prevented the attachment of the epidermis and was not suitable for skin regeneration [10]. The HHP method has been reported to be a superior method that does not damage the ECM and the HHP is also superior in terms of the processing time [11–16]. In this study, histological sections and SEM micrographs showed that pressurization at 200 MPa did not damage the collagen fibers and the elastic fibers of the pressurized skin, so in the next step, we will explore the response of pressurized skin in vivo to confirm this fact.

We used a WST-8 assay and an outgrowth culture to explore the viability of pressurized skin. Skin consists of an epidermis and a dermis, and the epidermis began to be removed after pressurization at 200 MPa. Therefore, we explored the outgrowth of the fibroblasts from the pressurized dermis in the outgrowth culture. Regarding the processing time of the HHP, there was no significant difference in the WST-8 assay in terms of the processing time for applying 200 MPa of pressure in both the NSS and the DW. This suggests that pressurization at 200 MPa for 5 min is sufficient for the inactivation of skin. However, the absorbance level upon being subjected to the pressure of 200 MPa for 5 min in the NSS showed some activity, but there was no significant difference when compared to that of the pressure of 100 MPa in the NSS; therefore, pressurization for 10 min would be desirable compared to a time of 5 min. Regarding the outgrowth of fibroblasts, the outgrowth was not observed in all of the NSS and DW groups at the pressure of 200 MPa. These results suggest that the outgrowth culture is a more accurate method to evaluate viability than the WST-8 assay because this assay detects the viability of cells only in the superficial part of the tissue and could not detect viable cells in the deeper part of the tissue. In this study, the DW could not inactivate cells in the deeper part as well as NSS, but it seemed to affect the elasticity of the skin. This indicates that the DW is not a suitable medium for performing HHP.

As presented above, we showed that pressurization at 200 MPa for only 10 min could inactivate the porcine skin as well as the cultured cells. The pressure of 200 MPa is a fairly low level compared with the HHP pressure of more than 600 MPa. The cold isostatic pressurization machine is large and expensive and used for pressurizations of up to 1000 MPa, but it is not portable. The inactivation method using the HHP is reported to be applied clinically with the purpose of disinfecting and devitalizing tissue containing vegetative microorganisms or tumor cells [15, 16]. One of the issues that needs to be resolved is the portability of the machine, because, at present, samples must be sent away for specimen analysis, and the procedure requires a secondary operation in order to use the pressurized tissue. However, if the target pressure could be as low as 200 MPa, the pressurization machine could be downsized and made portable and available in the operating room. In addition, the processing time of only 10 min is short enough to be used during a single operation.

Decellularized tissues have been developed and applied for use as allogeneic or xenogeneic tissues for patients' requiring tissue regeneration therapy. Using this HHP treatment, the autologous replantation of resected tissue containing tumor cells or infected tissue can be performed. In the field of skin surgery, large autologous skin grafting is needed for the treatment of benign skin tumors, such as giant congenital melanocytic nevi, or malignant skin tumors, such as squamous cell carcinoma or malignant melanoma [17–19]. If we could apply the HHP at the pressure of 200 MPa for the intraoperative inactivation of skin tumors, the removed skin could be reused as an autograft and we could avoid the harvesting of healthy skin from the patient's donor site. Therefore, this HHP method at the pressure of 200 MPa could be a promising treatment for skin tumors while simultaneously avoiding the harvesting of healthy skin tissue.

## 5. Conclusion

We showed that pressurization at 200 MPa for 10 min could inactivate porcine skin, which has a similar structure to human skin. This inactivation treatment could be a superior treatment for skin tumors that currently require the harvesting of large autologous skin grafts.
